# Localized rest and stress human cardiac creatine kinase reaction kinetics at 3 T

**DOI:** 10.1002/nbm.4085

**Published:** 2019-03-28

**Authors:** William T. Clarke, Mark A. Peterzan, Jennifer J. Rayner, Rana A. Sayeed, Mario Petrou, George Krasopoulos, Hannah A. Lake, Betty Raman, William D. Watson, Pete Cox, Moritz J. Hundertmark, Andrew P. Apps, Craig A. Lygate, Stefan Neubauer, Oliver J. Rider, Christopher T. Rodgers

**Affiliations:** ^1^ Oxford Centre for Clinical Magnetic Resonance Research (OCMR), Division of Cardiovascular Medicine RDM University of Oxford, John Radcliffe Hospital Oxford UK; ^2^ Wellcome Centre for Integrative Neuroimaging, FMRIB University of Oxford, John Radcliffe Hospital Oxford UK; ^3^ Department of Cardiothoracic Surgery, John Radcliffe Hospital Oxford University Hospitals NHS Foundation Trust Oxford UK; ^4^ Department of Cardiovascular Medicine University of Oxford, Wellcome Trust Centre for Human Genetics Roosevelt Drive Oxford UK; ^5^ Department of Physiology Anatomy University of Oxford Parks Road, Sherrington Building Oxford UK; ^6^ Wolfson Brain Imaging Centre University of Cambridge Box 65, Cambridge Biomedical Campus Cambridge UK

**Keywords:** ^31^P magnetic resonance spectroscopy, cardiac, creatine kinase, energy metabolism, phosphorus, saturation transfer, StreST, TRiST

## Abstract

Changes in the kinetics of the creatine kinase (CK) shuttle are sensitive markers of cardiac energetics but are typically measured at rest and in the prone position. This study aims to measure CK kinetics during pharmacological stress at 3 T, with measurement in the supine position. A shorter “stressed saturation transfer” (StreST) extension to the triple repetition time saturation transfer (TRiST) method is proposed. We assess scanning in a supine position and validate the MR measurement against biopsy assay of CK activity. We report normal ranges of stress CK forward rate (k_f_^CK^) for healthy volunteers and obese patients.

TRiST measures k_f_^CK^ in 40 min at 3 T. StreST extends the previously developed TRiST to also make a further k_f_^CK^ measurement during <20 min of dobutamine stress. We test our TRiST implementation in skeletal muscle and myocardium in both prone and supine positions. We evaluate StreST in the myocardium of six healthy volunteers and 34 obese subjects. We validated MR‐measured k_f_^CK^ against biopsy assays of CK activity.

TRiST k_f_^CK^ values matched literature values in skeletal muscle (k_f_^CK^ = 0.25 ± 0.03 s^−1^ vs 0.27 ± 0.03 s^−1^) and myocardium when measured in the prone position (0.32 ± 0.15 s^−1^), but a significant difference was found for TRiST k_f_^CK^ measured supine (0.24 ± 0.12 s^−1^). This difference was because of different respiratory‐ and cardiac‐motion‐induced B_0_ changes in the two positions. Using supine TRiST, cardiac k_f_^CK^ values for normal‐weight subjects were 0.15 ± 0.09 s^−1^ at rest and 0.17 ± 0.15 s^−1^ during stress. For obese subjects, k_f_^CK^ was 0.16 ± 0.07 s^−1^ at rest and 0.17 ± 0.10 s^−1^ during stress. Rest myocardial k_f_^CK^ and CK activity from LV biopsies of the same subjects correlated (*R* = 0.43, *p* = 0.03).

We present an independent implementation of TRiST on the Siemens platform using a commercially available coil. Our extended StreST protocol enables cardiac k_f_^CK^ to be measured during dobutamine‐induced stress in the supine position.

**Abbreviations used**
AHP: adiabatic half‐passage (pulse)
ATP: adenosine triphosphate
BMI: body mass index
CK: creatine kinase
CSI: chemical shift imaging
DANTE: delay alternating with nutation for tailored excitation
ECG: electrocardiogram
FAST: four‐angle saturation transfer
k_f_^CK^: creatine kinase pseudo first‐order forward rate
LV: left ventricular
PCr: phosphocreatine
StreST: stress‐saturation transfer
TRiST: triple repetition time saturation transfer
TwiST: two repetition time saturation transfer

## 1 INTRODUCTION

The rate and flux of the creatine kinase (CK) exchange mechanism have been shown to be sensitive measures of heart failure,^1^ which is a prevalent and burdensome disease.^2^, ^3^ The rate can be characterised by the pseudo first‐order forward rate constant, k_f_^CK^. Phosphorus magnetic resonance spectroscopy (^31^P‐MRS) enables noninvasive measurement of myocardial k_f_^CK^.^4^ In addition to measuring k_f_^CK^ in myocardium at rest, measuring k_f_^CK^ during pharmacologically induced stress would enable us to understand the effect of a perturbed CK mechanism in the stressed human heart.^1^, ^5^

Schär et al introduced the triple repetition time saturation transfer (TRiST) sequence to measure CK kinetics by ^31^P‐MRS at 3 T.^6^ A TRiST acquisition lasts 40 min (out of complete protocol totalling 84 min); it measures k_f_^CK^ in a one‐dimensional coronal stack of slices covering the heart and chest wall.^7^ 1D‐localised k_f_^CK^ measurement during inotropic stress was achieved by Weiss et al at 1.5 T using the four‐angle saturation transfer (FAST) and the derived “FASTest” method.^1^, ^4^ However, FAST and FASTest rely on small‐angle adiabatic pulses, which are not achievable at 3 T because of radiofrequency power requirements, power deposition and T_2_ relaxation.^8^ The 3 T TRiST protocol offers an established measurement technique which can be included in a larger cardiac ^1^H MR protocol on the same scanner. While 7 T ^31^P‐MRS has been used for 3D‐localised k_f_^CK^ measurements, the existing published methods have long duration and the higher field strength restricts the recruitment of subjects who have undergone surgical procedures.^9^

An increase in myocardial work can be reliably maintained, without physical exercise (and therefore motion), by administering dobutamine intravenously. However, the duration of intravenous infusion should be kept to a minimum length. Current guidelines prescribe 15 min stress protocols^10^ and they recommend patients should be scanned in the supine position, for safety in case of arrhythmia. Measuring k_f_^CK^ within this timeframe is currently only possible using the Two repetition time saturation transfer (TwiST) method, published during the course of this study.^7^ However, although TwiST assumes a fixed phosphocreatine (PCr) intrinsic longitudinal relaxation time (T_1_*), ie in the hypothetical case of PCr not undergoing chemical exchange, it is not yet known whether T_1_* remains constant in clinically relevant groups; eg obese, normal weight, and heart failure.

We therefore propose to perform stress k_f_^CK^ measurements at 3 T in two steps: first, derive a per subject baseline PCr T_1_* with TRiST; and second, perform two further scans during dobutamine‐induced stress to record the stress k_f_^CK^ in <20 min of dobutamine infusion. This stress saturation‐transfer (StreST) protocol yields a stack of 1D‐localised k_f_^CK^ measurements at rest and stress. We describe below the implementation of StreST and validate the underlying TRiST method in skeletal muscle, in human myocardium in the prone position and subsequently in the supine position, where we correlate surgical left ventricular (LV) biopsy‐obtained CK activity against preoperative myocardial k_f_^CK^ in a set of within‐patient paired measurements. We then demonstrate StreST by using it to record normative ranges of rest and stress k_f_^CK^ in the myocardium of normal volunteers and in older obese and age‐matched control cohorts.

## 2 THEORY

CK regenerates adenosine triphosphate (ATP) from PCr according to the equilibrium expression PCr^2−^ + MgADP^−^ + H^+^ ⇌ Cr + MgATP^2−^. CK provides the temporal energy reserve in muscle. TRiST uses steady‐state saturation of the terminal (γ) phosphate‐group of ATP to measure k_f_^CK^, the pseudo first‐order rate constant of the CK reaction in the forward (ATP‐generating) direction. Since the ^31^P nuclei in PCr and γ‐ATP are undergoing two‐site exchange, continuous saturation of γ‐ATP allows the forward exchange rate constant to be determined using

(1) $$k_{f}^{\mathrm{CK}}=\frac{1}{T_{1}^{\prime }}\left(1-\frac{M_{\mathrm{PCr}}^{\prime }}{M_{\mathrm{PCr}}^{\text{Ctrl}}}\right).$$

**Figure 1:**
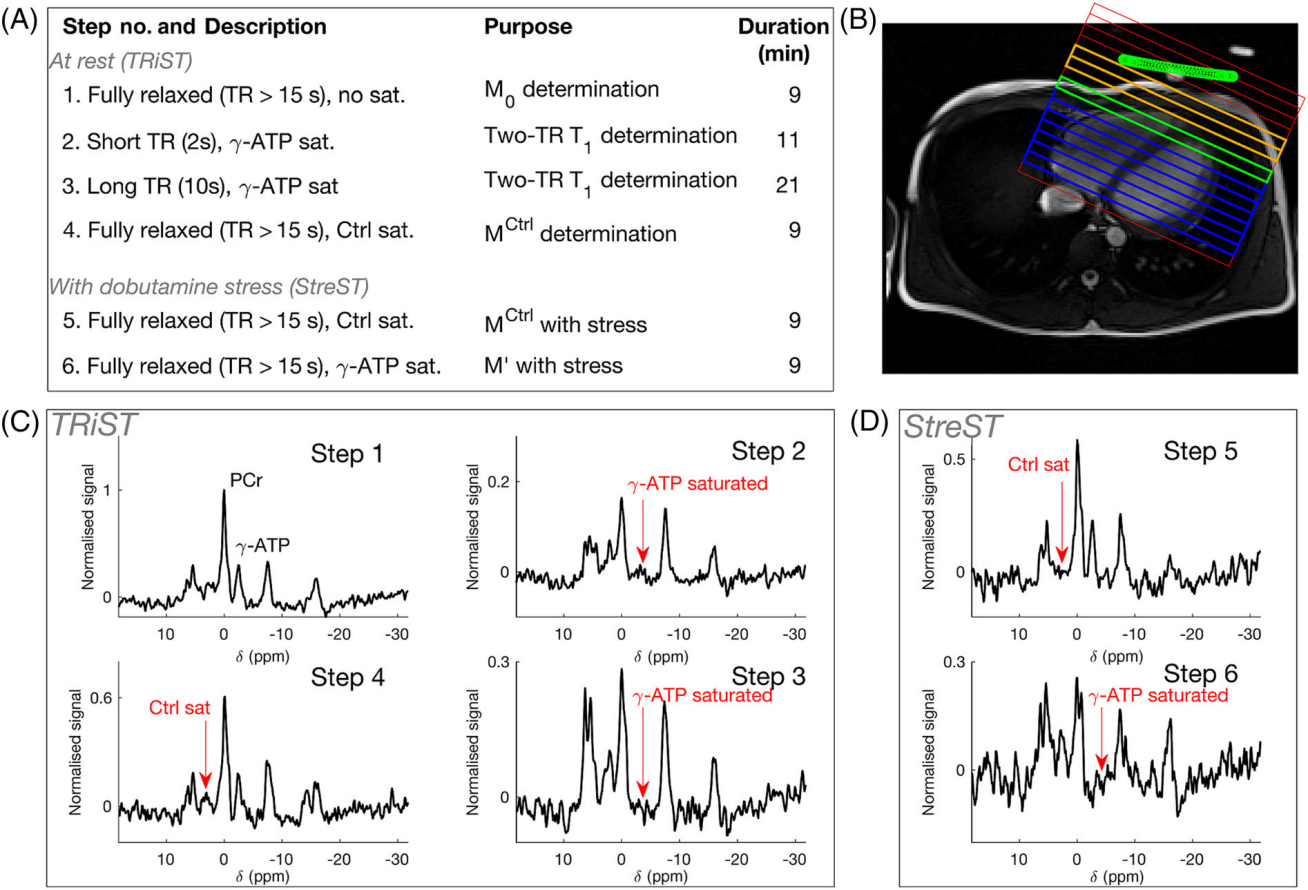
(A) Six‐step StreST protocol (including TRiST as steps 1–4); (B) 1H localiser with 1D CSI grid overlaid; the coil position is marked in green; orange = skeletal muscle, blue = myocardium, green = most anterior myocardial slice (coil slice distance ≤70 mm); (C) spectra acquired from the four steps of TRiST on a healthy, normal‐weight subject; (d) spectra acquired during dobutamine stress as part of the extension StreST measurement. In (C) and (D) signal is normalised to the PCr peak value in step 1

Table 1 summarises each parameter's physical meaning. TRiST measures $T_{1}^{\prime }$, $M_{\mathrm{PCr}}^{\prime }$and $M_{\mathrm{PCr}}^{\text{Ctrl}}$. This requires three steps: two to measure $M_{\mathrm{PCr}}^{\prime }$ and $T_{1}^{\prime }$ using the dual‐TR method,^8^ and a third to measure $M_{\mathrm{PCr}}^{\text{Ctrl}}$ (see Figure 1).

**Table 1:** Meanings of equation variables

**Symbol**	**Meaning**
*M*_0, PCr_	Equilibrium longitudinal magnetisation of PCr
$M_{\mathrm{PCr}}^{\text{Ctrl}}$	Measured steady‐state longitudinal PCr magnetisation, with mirrored control saturation applied
$T_{R}^{\text{Short}/\text{Long}}$	Short and long T_R_, where both $T_{R}^{\text{Short}/\text{Long}}$<5 T_1_
$M_{\mathrm{PCr}}^{\prime }$	Longitudinal PCr magnetisation, with on‐resonance saturation of γ‐ATP applied. *T*_R_ ≈ 5 *T*_1_
$M_{\mathrm{PCr}}^{\prime }\left(T_{R}^{\text{Short}}/T_{R}^{\text{Long}}\right)$	Measured steady‐state longitudinal PCr magnetisation, with on‐resonance saturation of γ‐ATP applied
$T_{1}^{\prime }$	Measured T_1_ in the presence of on‐resonance saturation of γ‐ATP applied
$T_{1}^{*}$	Intrinsic T_1_, ie without the effect of exchange

The intrinsic longitudinal relaxation time $T_{1}^{*}$, can be computed from^11^

(2) $$T_{1}^{*}=T_{1}^{\prime }\frac{M_{\mathrm{PCr}}^{\text{Ctrl}}}{M_{\mathrm{PCr}}^{\prime }}.$$

Therefore, by substituting Equation 2, Equation 1 may be recast in terms of $T_{1}^{*}$rather than $T_{1}^{\prime }$ 4:

(3) $$k_{f}^{\mathrm{CK}}=\frac{1}{T_{1}^{*}}\left(\frac{M_{\mathrm{PCr}}^{\text{Ctrl}}}{M_{\mathrm{PCr}}^{\prime }}-1\right).$$

$T_{1}^{*}$ is a hypothetical longitudinal relaxation constant for a molecule without chemical exchange. Assuming that $T_{1}^{*}$does not change from one scan to the next (eg in myocardium at rest vs under stress), then an additional measurement of k_f_^CK^ may be made in two steps: measuring $M_{\mathrm{PCr}}^{\text{Ctrl}}$ and $M_{\mathrm{PCr}}^{\prime }$. This is a similar assumption to that used in the FASTer/FASTest adaptation of the FAST method.^4^

### 2.1 StreST protocol

Our new StreST protocol comprises a rest measurement of k_f_^CK^ and PCr T_1_* using TRiST (four steps) and a measurement of k_f_^CK^ during intravenous pharmacological stress (two additional steps). The six (4 + 2) steps are shown in Table 2. All steps are completed in a single scanning session.

**Table 2:** Acquisition parameters for the StreST protocol. The first four steps are those of TRiST^6^; […] are parameters required for spill‐over correction of k_f_^CK^

**No.**	**θ**	**T**_**R**_ **(s)**	**Saturation target**	**Scan averages**	**Duration (min)**	**Measured parameters**	**TRiST**	**StreST**
1	90°	≥15	‐	2	9	*M*_0,PCr_, *M*_0,γ ‐ ATP_	✓	✓
2	90°	2 ( $T_{R}^{\text{Short}}$)	γ‐ATP	18	11	$M_{\mathrm{PCr}}^{\prime }\left(T_{R}^{\text{Short}}\right)$, $\left[M_{\gamma ‐\mathrm{ATP}}^{\prime }\left(T_{R}^{\text{Short}}\right)\right]$	✓	✓
3	90°	10 ( $T_{R}^{\text{Long}}$)	γ‐ATP	8	21	$M_{\mathrm{PCr}}^{\prime }\left(T_{R}^{\text{Long}}\right)$, $\left[M_{\gamma ‐\mathrm{ATP}}^{\prime }\left(T_{R}^{\text{Long}}\right)\right]$	✓	✓
4	90°	≥15	Control	2	9	$M_{\mathrm{PCr}}^{\text{Ctrl}}$, $M_{\gamma ‐\mathrm{ATP}}^{\text{Ctrl}}$	✓	✓
5	90°	≥15	Control	2	9	$M_{\mathrm{PCr}}^{\text{Ctrl}}$, $M_{\gamma ‐\mathrm{ATP}}^{\text{Ctrl}}$		✓
6	90°	≥15	γ‐ATP	2	9	$M_{\mathrm{PCr}}^{\prime }$, $\left[M_{\gamma ‐\mathrm{ATP}}^{\prime }\right]$		✓

In future studies using the proposed StreST protocol, subjects will be scanned in the supine position, instead of the prone position used in the original TRiST studies. Prone scanning is considered to be unsafe from a cardiac monitoring perspective, especially when scanning patients with heart failure who may be at greater risk of dangerous arrhythmia. One in 400 patients receiving dobutamine experience a life‐threatening arrhythmia.^12^

## 3 METHODS

All subjects were recruited in a manner approved by the local research ethics committee. All participants gave written informed consent.

### 3.1 Hardware, sequence and spectral analysis

All experiments used a 3 T TIM Trio MR scanner (Siemens, Erlangen, Germany). The scanner's body coil was used for ^1^H localisation. A 10 cm loop transmit‐receive surface coil (Pulse Teq, Chobham, UK) tuned to the phosphorus frequency was used for spectroscopy. The ^31^P coil was matched for each subject using an RF sweeper (Morris Instruments Inc., Ottawa, Canada). Coil loading was measured by inversion‐recovery on a phenylphosphonic acid/ethanol/chromium acetylacetonate fiducial fixed at the centre of the coil, as previously reported,^13^ and expressed as a reference voltage (the RMS RF voltage giving a 1 ms 180° pulse). The ^31^P coil was positioned above the apical myocardium of the subject. The positioning was checked using ^1^H localiser images of the heart and coil fiducial, and repositioning was carried out based on those images.

TRiST was implemented on the Siemens platform, following the reported description,^6^ as follows. The vendor's 1D chemical shift imaging (CSI) sequence was modified to continuously selectively saturate a chosen frequency while waiting to detect an R‐wave from an electrocardiogram (ECG) monitor attached to the subject. Once an R‐wave was detected saturation was continued for a further trigger delay until diastole, at which point an adiabatic half‐passage (AHP) excitation pulse, 1D phase encoding gradients and free‐induction‐decay readout were applied. Diastole was chosen to minimise myocardial motion.

The selective saturation was provided by a train of amplitude‐modulated delay alternating with nutation for tailored excitation (DANTE) pulses.^6^ B_1_‐insensitive 90^o^ excitation was provided by frequency‐cycled AHP pulses.^8^

Spectra were analysed by measuring the amplitude of the phased and apodized PCr peak relative to the baseline.^6^ Raw data from TRiST scans on the 3 T Achieva Philips scanner at Johns Hopkins were kindly supplied by Dr Schar and used to validate our fitting approach. $k_{f}^{\mathrm{CK}}$, $T_{1}^{\prime }$ and $T_{1}^{*}$ were calculated as described in the theory section (Equation 1) and as previously reported.^6^ The amount of direct (or spill‐over) saturation of PCr by the DANTE pulse (Q) was calculated as the ratio of $M_{\mathrm{PCr}}^{\text{Ctrl}}$/*M*_0, PCr_. (Q = 1 in the ideal case when there is no direct saturation, but only saturation via chemical exchange from saturated γ‐ATP.) Spectra from cardiac slices were selected using the transverse ^1^H localiser and analysed on a per‐slice basis.

### 3.2 Literature values

We surveyed the literature for values of k_f_^CK^ in human myocardium and skeletal muscle (Table 3). We used the arithmetic means of the literature k_f_^CK^ values and standard deviations (SD) for both tissues as a reference to validate our results.

**Table 3:** Literature values for human in vivo k_f_^CK^ in normal volunteers at rest

**Reference**	**Method** a	**Localisation** b	**Field (T)**	**N**	**Study mean ± SD or range (s** ^**−1**^ **)**
**Myocardium**					
1	FAST	1D‐CSI	1.5	16	0.32 ± 0.07
14	FAST	1D‐CSI	1.5	14	0.32 ± 0.06
15	FAST	1D‐CSI	1.5	15	0.33 ± 0.07
6	TRiST	1D‐CSI	3	8	0.32 ± 0.07
7	TwiST	1D‐CSI	3	12	0.33 ± 0.08
16	TDST	1D‐ISIS	3	15	0.32 ± 0.05
*Average*					0.323 ± 0.067
**Skeletal muscle (calf)**					
6	TRiST	1D‐CSI	3	6	0.26 ± 0.04
17	ST	‐	3	6	0.31 ± 0.04
17	ST	‐	7	6	0.35 ± 0.03
18	ST	TSE	3	30	0.23–0.29
19	Prog. Sat.	TSE	3	23	0.26–0.32
20	ST	1D‐ISIS	7	23	0.27–0.34
21	IT	‐	7	10	0.46 ± 0.09
22	IT	‐	7	7	0.26 ± 0.02
*Average*					0.274 ± 0.041

a FAST, four‐angle saturation transfer; TRiST, triple repetition time saturation transfer; TwiST, two repetition time saturation transfer; TDST, time‐dependent saturation transfer; ST, saturation transfer; Prog. Sat., progressive saturation; IT, inversion transfer.

b CSI, chemical shift imaging; ISIS, image‐selected in vivo spectroscopy; TSE, turbo spin echo.

### 3.3 Validation of TRiST implementation

#### 3.3.1 Skeletal muscle (calf)

We validated our TRiST implementation in the calf muscle of nine healthy volunteers (eight males, 30.6 ± 3.8 years old, 74.1 ± 11.3 kg). The subjects were positioned feet‐first‐supine in the scanner with the ^31^P loop coil under one leg. After ^1^H localisation, the 1D‐CSI grid was positioned running in the anterior–posterior direction. The protocol followed steps 1–4 in Table 2. Other parameters were: 160 mm field of view (FOV), 16 slices, 3 kHz bandwidth, 512 spectral points, and 200 V AHP transmit voltage (corresponding to 800 W peak power, and ~35 μT B_1_^+^ in vivo). Selective saturation of γ‐ATP and control saturation were achieved using 35 V DANTE pulses (corresponding to 24.5 W peak power, and ~6 μT B_1_^+^ in vivo).

#### 3.3.2 Myocardium in prone position

Ten healthy volunteer subjects (six males, 29.6 ± 4.9 years old, 70.7 ± 18.2 kg) were scanned using the newly implemented TRiST protocol (steps 1–4, rest k_f_^CK^ only) to measure myocardial k_f_^CK^. The scans were completed in the prone position as per previously published methods.

CSI acquisition parameters were as follows: 160 mm FOV, 16‐step matrix, 3 kHz bandwidth, 512 samples. The CSI grid was positioned perpendicular to a transverse localiser covering the heart, with the CSI delineated dimension aligned coronally (parallel to the band of skeletal muscle lying between the coil and the heart). The AHP transmit voltage was 210 V (ie 882 W peak power), and the amplitude‐modulated DANTE voltage was maximised within the constraints of the specific absorption rate (SAR) for the short TR scan (typically to ~30 V, ie 18 W peak power). Spectra from cardiac slices were selected using the transverse ^1^H localisers for analysis as described above. The data from the most apical slice containing only myocardium and blood (but not skeletal muscle) were also analysed separately. The coil to slice distance was <60 mm for these slices.

#### 3.3.3 Myocardium in supine position

As detailed above, the full StreST protocol will include administering intravenous dobutamine, for which it is preferred to position the subject supine. To test whether the change of position (prone to supine) affects the initial TRiST measurement in the StreST protocol, we scanned the same 10 subjects as used in the previous section (six males, 29.6 ± 4.9 years old, 70.7 ± 18.2 kg) again. This time, scans were in the supine position, using the newly implemented TRiST protocol (steps 1–4, rest k_f_^CK^ only). Other acquisition and analysis parameters were identical to the previous section.

### 3.4 Effect of intra‐scan B_0_ fluctuation

The potential effects of respiratory and cardiac motion induced B_0_ changes on TRiST $k_{f}^{\mathrm{CK}}$values were analysed using Bloch simulations of the full TRiST protocol. A dual‐echo CINE gradient echo sequence was used to measure the range of B_0_ values present in the un‐shimmed apical myocardium of a single subject in different cardiac phases and respiratory states in both supine and prone positions. A sinusoidal frequency sweep with amplitude of 0, 20, 40, 60 and 80 Hz was applied to the Bloch simulation to simulate respiratory motion. The AHP pulse was simulated with three different $B_{1}^{+}$ magnitudes: 12, 23 and 35 μT, and the DANTE saturation pulse was scaled appropriately to simulate the experiment. Simulations were run with 10 000 repetitions, each having a random initial cardiac and respiratory phase. Each independent step of TRiST was simulated and combined to give a measured k_f_^CK^. Simulation parameters were taken from Table 1 (heart muscle) in reference 23 with k_f_^CK^ varied from 0.1–0.5 s^−1^. SNR was calculated for PCr and γ‐ATP, and the simulation was scaled so the PCr SNR in step 4 was equal to 15.

### 3.5 Validation of MRS measured k_f_^CK^ by surgical biopsy

In a cohort of 25 subjects listed for clinically indicated surgery for either severe aortic stenosis with preserved (*n* = 18) or impaired (*n* = 4) left ventricular ejection fraction (LVEF ≥ or < 55%, respectively), severe primary mitral regurgitation (*n* = 2), or atrial myxoma (n = 1), $k_{f}^{\mathrm{CK}}$ was measured by supine TRiST and compared with CK activity measured ex vivo from surgical LV biopsies.

All subjects preoperatively underwent the TRiST MRS protocol in the supine position as described in the previous section. k_f_^CK^ was measured for the most apical voxel identified as purely myocardium on ^1^H localisers. Intra‐operative biopsies from LV septal endocardium were obtained by the operator 10–15 min after cardiopulmonary bypass was established, then immediately placed into liquid nitrogen and stored at −80°C.

For the measurement of CK activity, a heaped spatula full of frozen, crushed LV tissue was combined with CK‐NAC reagent (Thermo Fisher Scientific catalogue code TR14010) and the prescribed series of reactions were monitored using a spectrophotometer to measure the absorbance of NADH at 340 nm and 37°C over 3 min.^24^, ^25^, ^26^ CK activity (IU/mL) was calculated from the rate of change in absorbance of NADH, corrected for reaction volume and an assay‐specific correction factor, averaged over three runs and normalised to Lowry protein (mg/mL). Results are presented as CK activity (IU/mg protein). The MRS‐measured CK rate constant was then correlated with biopsy‐measured CK activity.

### 3.6 Validation of the stress k_f_^CK^ measurement (StreST) in healthy volunteers

The validity of the final reduced‐time $k_{f}^{\mathrm{CK}}$ measurement (from steps 5 and 6 of the full StreST protocol) was tested in six healthy volunteers (all males, 31 ± 9 years old, 75 ± 8 kg). After the initial TRiST measurement (steps 1–4), the follow‐on measurement (steps 5 and 6) was made without repositioning and with the subject still at rest (ie a “null stress” control condition). The PCr‐matched filtered signal‐to‐noise ratio (SNR) of the control acquisition (step 4 in Table 2) was determined. The $k_{f}^{\mathrm{CK}}$, $T_{1}^{\prime }$, $T_{1}^{*}$ and Q were reported.

Reproducibility of the PCr amplitude of individual scans was assessed from the fourth and fifth scans, which were acquired with identical protocols in this validation step (ie corresponding to rest and dobutamine‐stress scans in patients). Two methods of measuring $M_{\mathrm{PCr}}^{\prime }$were compared: (i) by saturation‐correction in TRiST; and (ii) directly from the sixth StreST step (see Table 2). The correlation and Bland–Altman statistics for these two $k_{f}^{\mathrm{CK}}$measurements were computed.

### 3.7 StreST in obese subjects and age‐matched controls

As many cardiac patients are obese, to allow the measurement to be validated in a real‐world population, the full StreST protocol (steps 1–6), including dobutamine‐induced stress during the second measurement, was performed in age‐matched obese and normal‐weight volunteers. StreST data were acquired from an obese cohort (*N* = 18, 5 males, 13 females, aged 49 ± 13 years old, body mass index (BMI) = 35 ± 5 kg/m^2^), and a normal‐weight control cohort (*N* = 6, one male, five females, aged 53 ± 22 years old, BMI = 24 ± 2 kg/m^2^). TRiST alone (steps 1–4) was run in 10 further normal‐weight volunteers (seven males, three females, 40 ± 21 years old, BMI = 23 ± 3 kg/m^2^).

For stress scans, dobutamine was administered intravenously, starting at 5 μg kg^−1^ min^−1^, and increasing the infusion rate every 3 min until a target heart rate of 65% maximum heart rate [ie 220 (age in years)] was achieved; this target heart rate was then maintained at a steady state for ~18 min while the additional StreST measurements (steps 5–6) were made. Spectra from cardiac slices were selected using the transverse ^1^H localisers for analysis as described above. The data from the most apical slice containing only myocardium and blood (but not skeletal muscle) were also analysed separately. The coil to slice distance was ≤70 mm for these slices.

## 4 RESULTS

### 4.1 Literature values

The results of the survey of literature k_f_^CK^ are provided in Table 3. The inter‐study mean ± SD k_f_^CK^ values were 0.27 ± 0.04 s^−1^ (skeletal muscle) and 0.32 ± 0.07 s^−1^ (myocardium).

### 4.2 Validation of TRiST implementation

#### 4.2.1 Skeletal muscle (calf)

**Figure 2:**
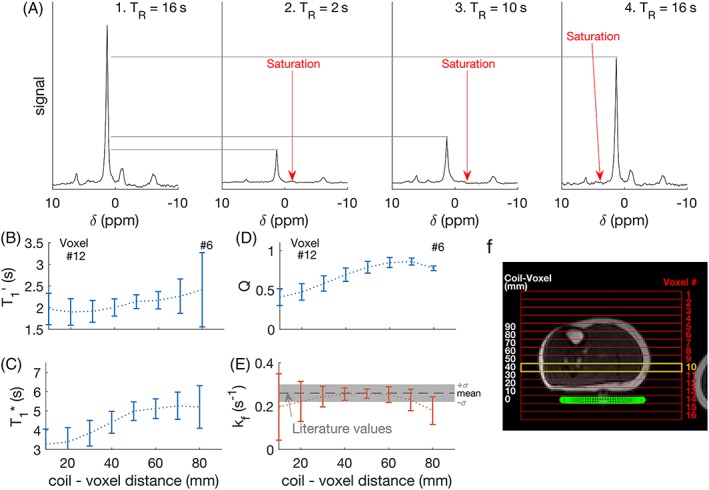
(a) Spectra from the four constituent scans of TRiST, showing the site of selective saturation, taken from a single slice in one subject (number 10, marked in orange in (f); (b) saturation‐affected T_1_ (T_1_’) for each subject in each slice, plotted as a function of distance from the coil. Error bars indicate (mean ± SD); (c) shows the intrinsic T_1_ (T_1_*), (d) the amount of direct PCr saturation (Q), (e) the k_f_^CK^, and (f) shows a localiser with a CSI grid overlaid (red), the slice plotted (orange), and the coil position (green)

In all subjects, seven or more slices were identified in the transverse ^1^H localiser images as containing mainly skeletal muscle. The mean (± SD) PCr SNR in the control saturation acquisition (step 4) was 45 ± 32. Example spectra are shown in Figure 2a.

Consistent $T_{1}^{\prime }$ and $k_{f}^{\mathrm{CK}}$ values were found across the five slices corresponding to 20–70 mm from the coil in all subjects. The average $T_{1}^{\prime }$in these slices was 2.2 ± 0.4 s, and $k_{f}^{\mathrm{CK}}$was 0.25 ± 0.03 s^−1^. In the two slices furthest from the coil (~70–80 mm), which also contained the tibia and the highest amount of subcutaneous fat, $T_{1}^{\prime }$ was higher and $k_{f}^{\mathrm{CK}}$lower (Figure 2b,e). $T_{1}^{*}$ was less consistent across slices and between the subjects (Figure 2c).

Complete saturation (>95% saturation) of γ‐ATP was observed in all subjects, and in all slices except the two furthest from the coil; in these slices the residual γ‐ATP level was 12 ± 3% of the control saturation scan. The ratio of the control‐saturation PCr peak to the no‐saturation PCr peak (Q, a measure of direct saturation of PCr by DANTE) was >0.5 for depths from 30–80 mm (Figure 2d). In the closest slices to the coil (10 and 20 mm) Q was <0.5, ie substantial direct saturation occurred.

Results from this subsection and others are summarised in Table S1.

#### 4.2.2 Myocardium in prone position

From the 10 healthy volunteers scanned in the prone position, 29 slices were identified as corresponding to myocardium in the transverse ^1^H localisers and had sufficient SNR for analysis (PCr SNR >10 in the control scan).

The all‐slice mean ± SD k_f_^CK^ was 0.29 ± 0.09 s^−1^. Analysing only the most anterior purely myocardial slice in each subject (10 slices) gave a mean k_f_^CK^ of 0.32 ± 0.15 s^−1^.

The all‐slice mean T_1_^’^ was 2.7 ± 1.0 s and T_1_^*^ was 4.7 ± 1.6 s. The mean (± SD) PCr SNR was 18 ± 8. Analysing only the most anterior purely myocardial slice in each subject gave SNR = 19 ± 5, T_1_^’^ = 2.9 ± 0.6 s, and T_1_^*^ = 5.2 ± 0.8 s.

#### 4.2.3 Myocardium in supine position

The same 10 healthy volunteers were also scanned in the supine position. In this dataset, 30 slices were identified as corresponding to myocardium in the transverse ^1^H localisers and had sufficient SNR for analysis.

The all‐slice mean ± SD k_f_^CK^ was 0.15 ± 0.10 s^−1^. Analysing only the most anterior purely myocardial slice in each subject gave a mean k_f_^CK^ of 0.24 ± 0.12 s^−1^.

The all‐slice T_1_^’^ was 2.5 ± 1.1 s, and T_1_^*^ was 4.4 ± 1.9 s. The mean (± SD) PCr SNR was 16 ± 9. Analysing only the most anterior purely myocardial slice in each subject gave SNR = 17 ± 6, T_1_^’^ = 2.3 ± 0.5 s, and T_1_^*^ = 4.6 ± 1.0 s.

### 4.3 Effect of intra‐scan B_0_ fluctuation

**Figure 3:**
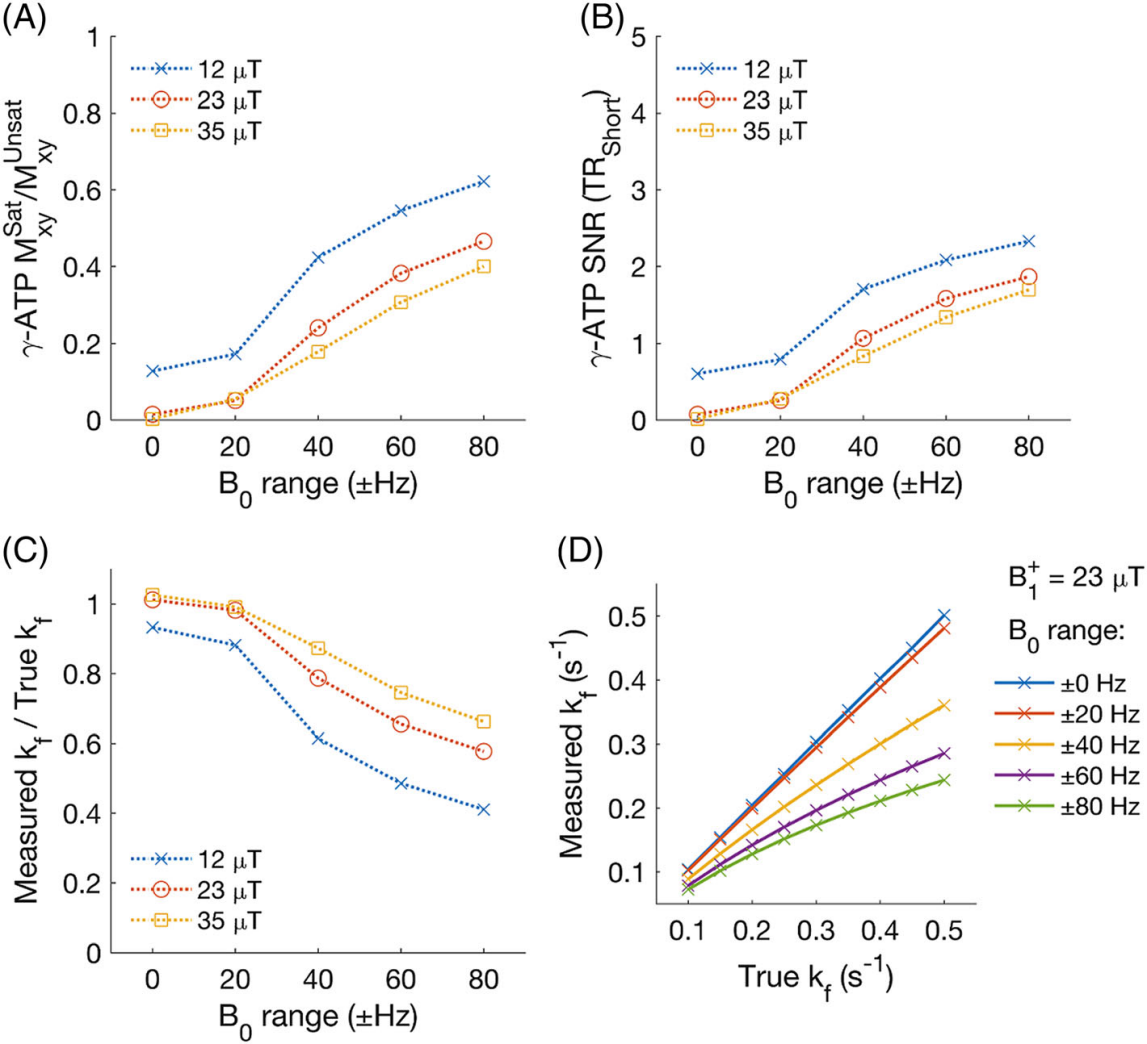
Simulated effect of respiration on the measurement of k_f_^CK^. (a) the ratio of γ‐ATP transverse magnetisation in the presence of steady‐state saturation (with respiration‐induced B_0_ variation) versus the same sequence with no steady‐state saturation. (b) the residual γ‐ATP peak SNR. (c) the ratio of measured k_f_^CK^ to true (simulation) k_f_^CK^. True k_f_^CK^ = 0.30 s^−1^. (d) Measured k_f_^CK^ in the presence of respiration‐induced B_0_ variation at different values of true k_f_^CK^

The single subject measurement of B_0_ established that the mean range of γB_0_ experienced in the apical myocardium due to cardiac motion in a ^31^P experiment is 34.3 Hz (supine) and 34.6 Hz (prone), and due to respiratory motion is 66.7 Hz (supine) and 36.1 Hz (prone). (Figures S1 and S2). As the range of B_0_ variation was increased in the simulations, the amount of time during the DANTE saturation pulse when M_z,γ‐ATP_ = 0 decreased (ie γ‐ATP saturation was not achieved at all times), even though the SNR of the residual γ‐ATP peak in TRiST steps 2 and 3 remained very low: SNR <2.5 (Figure 3a,b). With increasing B_0_ fluctuation amplitude and decreasing γ‐ATP saturation, the measured k_f_^CK^ also decreased (Figure 3c). At the level of the estimated B_0_ variation due to respiration in our study, the measured k_f_^CK^ was simulated to be 0.61 times the true k_f_^CK^ in a supine position and 0.85 times the true k_f_^CK^ in the prone position. The linearity of the ratio of measured k_f_^CK^/true k_f_^CK^ decreases with increasing B_0_ variation (Figure 3d).

### 4.4 Validation of MRS measured k_f_^CK^ by surgical biopsy

For the 25 subjects listed for clinically indicated surgery, mean (± SD) k_f_^CK^ was 0.21 ± 0.10 s^−1^ and mean (± SD) biopsy‐measured CK activity was 3.96 ± 1.70 IU mg^−1^ protein. The Pearson's Linear Correlation Coefficient (Pearson's R) was 0.43 with a statistically significant correlation (*p* = 0.03).

### 4.5 Validation of the stress k_f_^CK^ measurement (StreST) in healthy volunteers

**Figure 4:**
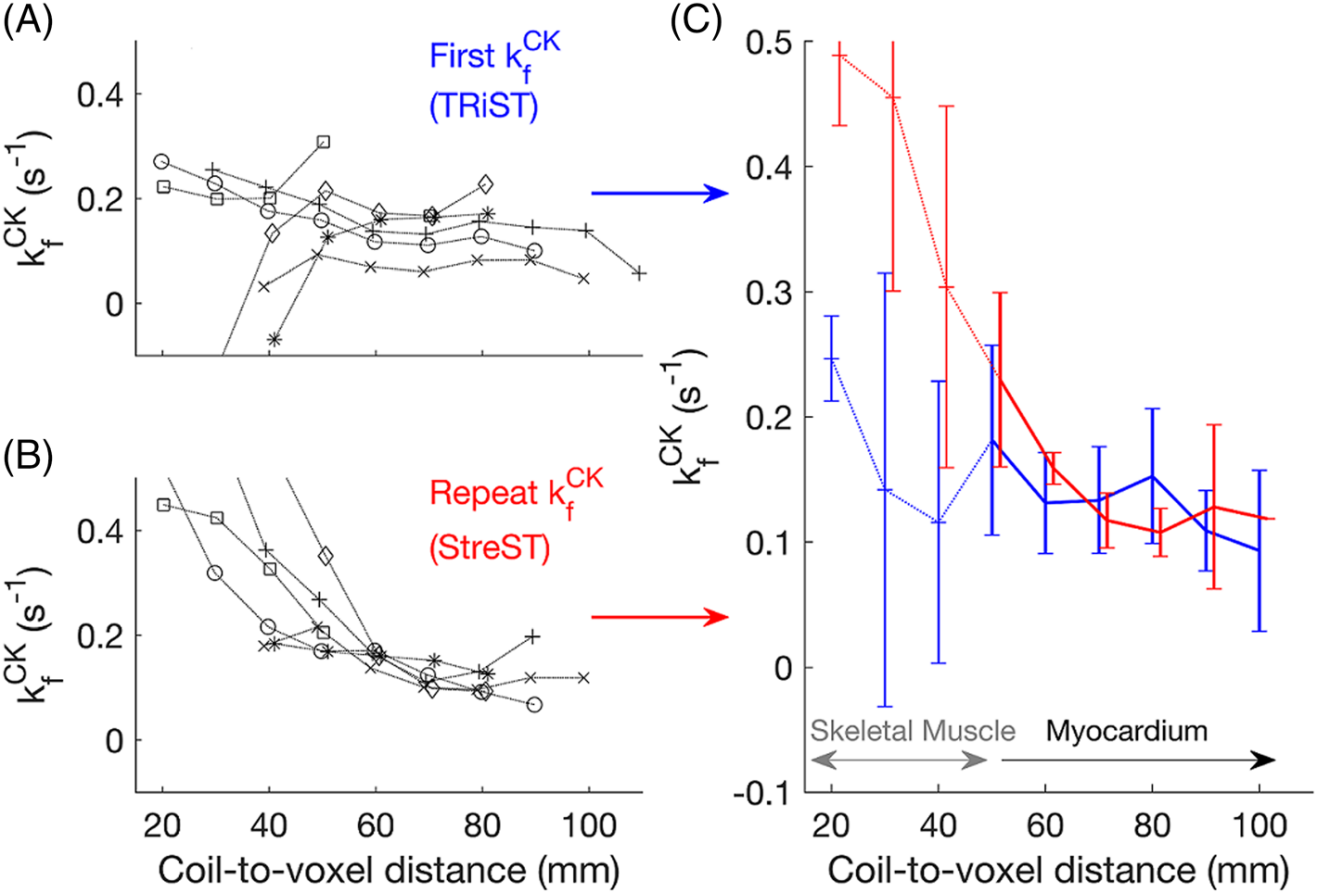
(a) TRiST and (b) StreST measured k_f_^CK^ in the chests of six normal volunteers. Results are plotted as a function of coil‐slice distance. StreST was performed without dobutamine stress for this validation study. Different markers denote different subjects. In (c) the inter‐subject mean and SD k_f_^CK^ is shown for TRiST (blue: Also the first^t^ measurement of StreST) and the second measurement of StreST (red)

**Figure 5:**
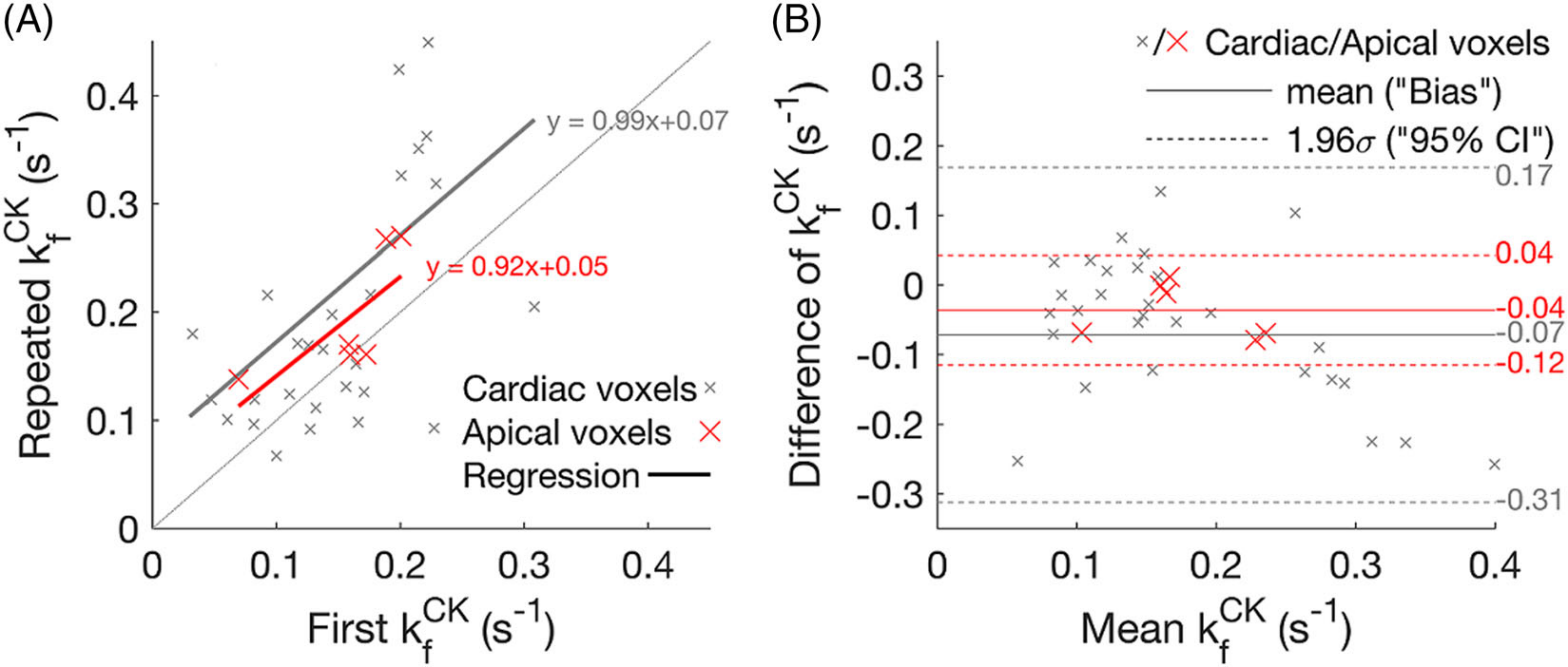
(a) Per‐slice correlation plot of the two k_f_^CK^ measurements in StreST (first measurement equivalent to TRiST). All myocardial slices are shown, with the most apical cardiac slice for each subject shown in red. Results of linear regressions are also shown. (b) Bland–Altman comparison of the two k_f_^CK^ measurements. The bias and 95% confidence intervals for each set of slices (all cardiac and apical) are marked

All the per‐subject and mean k_f_^CK^ values from the myocardial and skeletal muscle voxels of the six healthy volunteer rest‐rest (“null stress” control) StreST scans are plotted in Figure 4. In these scans, 36 slices were identified as corresponding to myocardium in the transverse ^1^H localisers and had sufficient SNR for analysis (PCr SNR >10 in the control scan). The all‐slice mean (± SD) PCr SNR was 16 ± 9, T_1_^’^ was 2.9 ± 1.0 s, and T_1_^*^ was 4.8 ± 1.8 s. The all‐slice mean k_f_^CK^ of the first measurement (TRiST) was 0.14 ± 0.08 s^−1^ and the all‐slice mean of the second measurement (dobutamine was not administered for this validation experiment) was 0.22 ± 0.14 s^−1^. A per‐slice comparison of these k_f_^CK^ measurements yielded a correlation of 0.51 (Figure 5a). Bland–Altman (Figure 5b) analysis yielded a bias of −0.08 s^−1^ with 95% confidence intervals (CIs) of +0.16 s^−1^ and − 0.31 s^−1^. A paired Student's t‐test showed statistical significance between the two measurements (*p* = 0.0006).

Analysing only the most anterior purely myocardial slice in each subject (six slices) gave SNR = 15 ± 5, PCr T_1_^’^ = 3.0 ± 0.6 s, PCr T_1_^*^ = 5.7 ± 0.9 s, k_f_^CK^ (first) = 0.18 ± 0.08 s^−1^, k_f_^CK^ (second) = 0.18 ± 0.05 s^−1^, and a per‐slice correlation of 0.62. The mean coil‐to‐voxel distance for these slices was 53 ± 7 mm. Bland–Altman (Figure 5b) analysis yielded a bias of −0.04 s^−1^ with 95% CIs of +0.04 s^−1^ and − 0.12 s^−1^. A paired Student's t‐test showed no statistical significance between the two measurements (*p* = 0.11).

Further reproducibility measurements are presented in the supporting information. The comparison of the PCr amplitudes of the fourth and fifth steps yielded a correlation of 0.99 (Figure S3a,b). The comparison of the two methods of calculating M_0_’ yielded a correlation of 0.96 (Figure S3c,d).

The coil reference voltage, measuring the degree of coil loading, varied by <10% for all six subjects, and was within 25% of the values measured in the skeletal muscle validation.

### 4.6 StreST in obese subjects and age‐matched controls

In both obese and normal‐weight volunteers (34 in total) the average k_f_^CK^ in all myocardial slices (with PCr SNR >10) was 0.12 ± 0.08 s^−1^. The average PCr SNR was 14 ± 9 across the 209 slices analysed.

**Figure 6:**
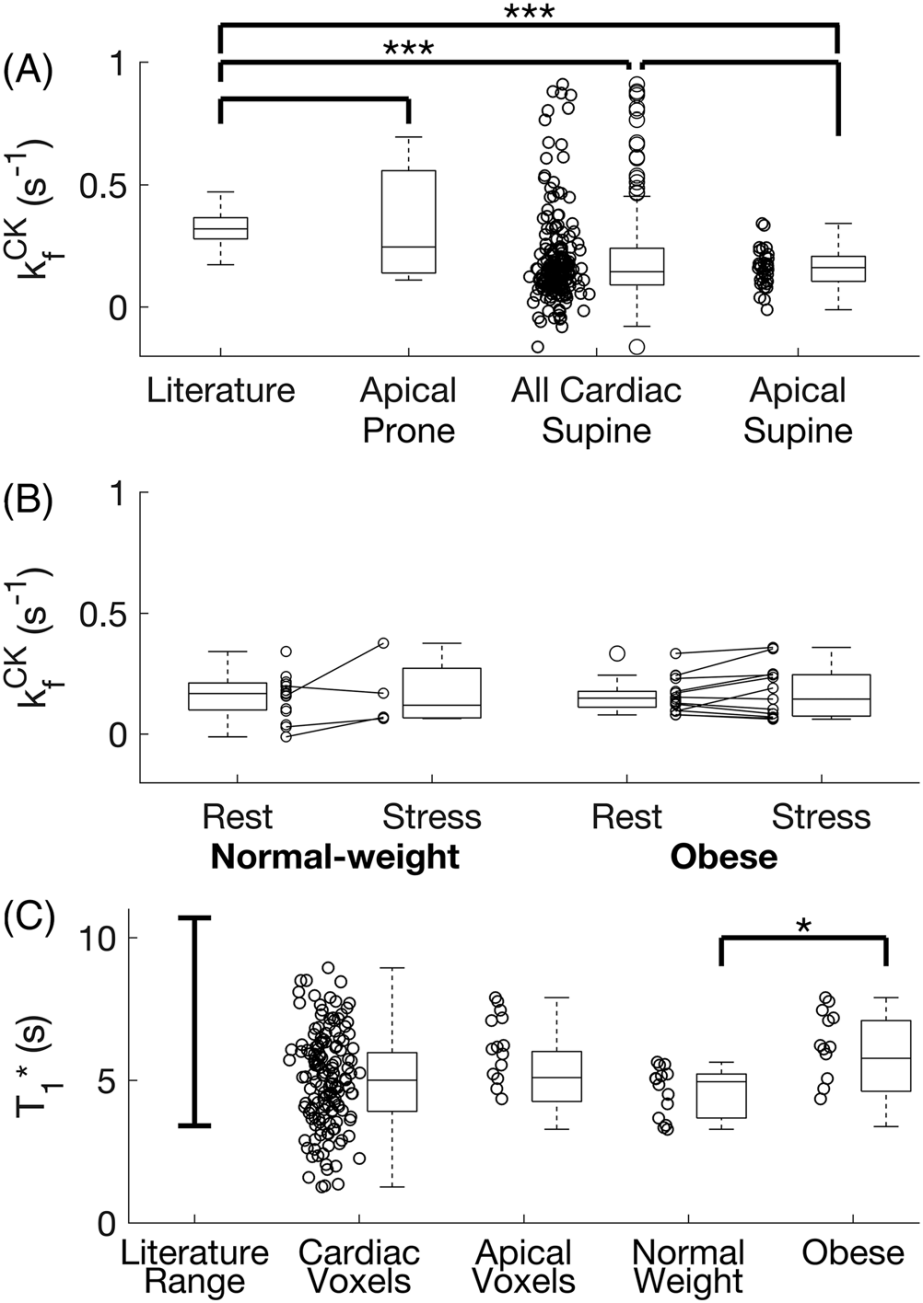
(a) The measured k_f_^CK^ in all subjects undergoing the 4 scan TRiST measurement. Shown are the results from the prone validation, and all myocardial slices and anterior myocardial slices from supine scans. (b) Rest and stress measurements from the selected slices of 34 normal‐weight and obese volunteers. Negative values of k_f_^CK^ are shown in this plot. While negative k_f_^CK^ values are not physically meaningful, they arise from noise entering into equation 1. (c) Reported literature range of intrinsic T_1_ (T_1_*)^7^ compared with that measured in this study, for all cardiac slices, the most apical cardiac slices, and the apical cardiac slices from normal‐weight and obese subjects

Analysing only the most anterior myocardial slice of each subject (34 slices), k_f_^CK^ was 0.16 ± 0.08 s^−1^ (Figure 6a). The average PCr SNR was 15 ± 6.

In the subjects that underwent both rest and stress measurements, the mean k_f_^CK^ at rest was 0.16 ± 0.07 s^−1^ (obese) and 0.15 ± 0.09 s^−1^ (normal weight). Under stress the values were 0.17 ± 0.11 s^−1^ (obese) and 0.17 ± 0.15 s^−1^ (normal weight). This data is shown in Figure 6b.

The T_1_* of the two cohorts was 5.69 ± 1.43 s for obese and 4.67 ± 1.92 s for normal‐weight subjects (Figure 6c); this difference is statistically significantly (Student's t‐test, *p* = 0.02).

## 5 DISCUSSION

We have implemented a new StreST protocol for measuring human CK rate constants in the human heart during dobutamine‐induced stress. In so doing, we have also implemented the published TRiST protocol measuring k_f_^CK^ at rest for the first time on a Siemens scanner, and using a commercially available coil. We have tested StreST (and hence also TRiST) in calf and cardiac muscle and applied it in the hearts of normal volunteers and obese subjects. We have demonstrated a correlation between our MRS measured value of k_f_^CK^ and CK activity in human LV biopsies.

Measurements in calf muscle show that our implementation of TRiST measures k_f_^CK^ are in line with literature values up to 70 mm from coil. The coil loading changed by up to 25% between skeletal muscle and the thorax. Therefore, we expected accurate myocardial measurement in cardiac slices ≤70 mm from the coil, ie we expected that k_f_^CK^ in apical cardiac slices could be measured robustly. This is corroborated by a Monte Carlo propagation of error analysis (Figure S4), which suggests the precision and accuracy of the technique is acceptable for PCr SNR >10. Only apical myocardial slices achieve this SNR level consistently in all subjects. The working depth of the TRiST protocol could be improved by a different choice of coil: for instance, a different design of transmit coil (eg a larger loop or two loops in quadrature) would ensure effective saturation and excitation at greater depths. A receive array might also be used for signal reception to improve SNR, although this might come at the expense of greatly increased signal contamination by nonmyocardial tissue because spatial localisation in TRiST is reliant on a restricted sensitivity profile of the coil in two dimensions.

Myocardial k_f_^CK^ measured in the prone position further validated the new implementation of TRiST with all cardiac slices in 10 subjects giving 0.29 ± 0.09 s^−1^ and k_f_^CK^ from only the most apical voxel for each subject giving a mean of 0.32 ± 0.15 s^−1^, although the SD of this measurement is double that reported in the literature (Table 3). In the supine position, the measured k_f_^CK^ throughout this study is much lower than the paired prone estimate, the literature estimate of 0.32 s^−1^, and our own 7 T k_f_^CK^ estimate (0.35 ± 0.05 s^−1^).^9^ It is therefore likely that the absolute value of k_f_^CK^ measured in a supine position is an underestimate. However, simulations of the effect of B_0_ variation during respiratory and cardiac cycles and correlation with biopsy‐measured CK activity in 25 patients indicate that despite the low absolute value of supine MRS‐measured k_f_^CK^, trends in our measured k_f_^CK^ values are still meaningful, that is, increases or decreases in measured k_f_^CK^ correspond to real increases or decreases.

We invested considerable effort in studying the possible causes of the lower supine TRiST k_f_^CK^ measurements. A thorough validation of the sequence timings was performed in the vendor simulation environment and by capturing the live waveforms of the triggered sequence using a digital oscilloscope on the scanner. Data shared from Johns Hopkins were used to validate our analysis process, which performed comparably with the Johns Hopkins analysis. Bloch simulations of the TRiST method indicated that if constant steady‐state saturation of γ‐ATP is not maintained completely throughout the mixing time, the measured k_f_^CK^ will underestimate the true k_f_^CK^ by a predictable scaling that is approximately linear for modest B_0_ fluctuation amplitudes (Figure S5). Note that this effect can occur even when the γ‐ATP peak is well suppressed in the observed saturated spectra. It is proposed that this is produced by B_0_ shifts, due to respiration or cardiac motion, intermittently shifting the γ‐ATP resonance away from the target selective saturation frequency. We have shown that the range of B_0_ experienced in the myocardium is raised in this experiment when the subject is supine rather than prone (~60 Hz range vs 30 Hz). The choice of supine scanning was necessitated in this study for subject safety during pharmacological stress and will be required in our institute for further studies using StreST in patients with established cardiac diseases. Scanning supine also helps coil placement and matching.

The effect of B_0_ shifts due to respiration was found, by simulation, to decrease the measured k_f_^CK^ by ~1.6 times for supine scans. This factor was found to be constant for all values of k_f_^CK^ as long as the B_0_ shifts did not exceed a range of 80 Hz. Above this level the effect is nonlinear, decreasing the sensitivity of TRiST to changes in k_f_^CK^. Our simulations also suggest that even in the prone position the true value of k_f_^CK^ is likely to be underestimated by the TRiST method. At the measured amplitude of B_0_ fluctuation the correction remains mostly linear and so relative changes in k_f_^CK^ are preserved for both prone and supine scanning.

StreST reduces the time of the consecutive measurement from 40 min to 20 min by assuming that the subject's T_1_* is constant, which makes it feasible to measure k_f_^CK^ during dobutamine infusion at 3 T. Previously, Weiss et al used an adaptation of the FAST protocol to measure k_f_^CK^ during stress in 13 min at 1.5 T.^1^ The validation of StreST applied without dobutamine showed that the method is able to reliably measure the same k_f_^CK^ in a reduced duration in the most apical, high SNR voxels. It is therefore likely that the assumption of static between‐scan T_1_* is reasonable. In voxels with low SNR or experiencing high direct saturation (low Q, eg skeletal muscle) the reduced duration measurement does not match the full TRiST measurement and introduces high variance.

The average PCr T_1_* calculated, as per Equation 2, was different for the two cohorts: normal‐weight and obese (*p* = 0.02). This suggests that to accurately measure stress k_f_^CK^, T_1_* must either be determined per subject, as in StreST, or per cohort in a pilot study designed to measure T_1_*. We do not recommend assuming a single PCr T_1_* for all human subjects.

Like TRiST, StreST has diagnostic potential for noninvasively assessing the CK system and by extension a subject's contractile reserve.^27^ Sensitivity to contractile reserve would be valuable in patients who are not able to undergo conventional stress testing, eg severe valvular heart disease. The CK system is also a major mechanism for controlling cytosolic [ADP]. A raised cytosolic [ADP] at stress contributes to increased LV end‐diastolic pressure and diastolic dysfunction.^28^ A raised LV end‐diastolic pressure is characteristic of heart failure with preserved ejection fraction, which comprises approximately half of all clinically presenting heart failure cases.

Cardiac positron emission tomography (PET) can also measure myocardial metabolic reaction kinetics through the uptake of tracers.^29^ It is able to confirm viability in suspected hibernating myocardium using glucose tracers.^30^ PET is able to detect uptake in ingressing inflammatory cells and has emerging roles in the detection of prosthetic valve endocarditis^31^ and inflammatory atherosclerotic coronary and carotid plaques.^32^, ^33^ However, the onward metabolism of the tracer after uptake cannot be assessed and it is not possible to distinguish which cell type is responsible using PET alone. The MRS technique presented here is specific to CK‐expressing cells, ie cardiomyocytes. Cardiac MR(S) and PET measure similar information with differing trade‐offs in temporal and spatial resolution. The use of both in tandem could offer complementary information.^30^

StreST was demonstrated in a control cohort, as well as an obese cohort. The TRiST component of the protocol was run successfully on 34 out of 35 initial subjects. The full StreST protocol was completed by 17 out of 24 subjects, with five subjects electing not to complete due to discomfort, and two scans were stopped after exceeding the local limit on maximum scan duration. The mean ± SD time of a complete StreST protocol was 103 ± 7 min; steps 1–6 of StreST take 64 min in total. The TRiST and StreST techniques are being applied in ongoing studies, building on the initial cohort scans in this work.

## 6 CONCLUSION

In this work, we introduced an extended StreST protocol that enables measurement of k_f_^CK^ during a 20‐min dobutamine infusion at 3 T. We also independently implemented the TRiST protocol on a Siemens 3 T scanner using commercially available hardware. We compared TRiST measured in the prone and supine position and provided a non‐MR validation of MR‐measured k_f_^CK^. We showed by simulations that respiratory‐induced motion can lead to incomplete γ‐ATP saturation during the saturation‐transfer phase of the TRiST sequence, even in the case where the γ‐ATP peak is absent from the saturated spectra. Linear correction can compensate for these effects for light to moderate B_0_‐field fluctuation amplitudes.

## Supporting information

Figure S1 ‐ B_0_ variation in apical myocardium due to cardiac and respiratory motion in a **supine** position. **a** Mean(±SD) of per‐voxel B_0_ values in the apical myocardium of one subject. The B_0_ field was measured at different phases of the cardiac cycle and at three respiratory positions (inhaled, exhaled and “middle”). **b** Range of B_0_ values experienced at each cardiac phase across the three respiratory positions. The plot shows the range of the means (black) and the maximum range (red), corresponding to the first standard deviation of the distributions. In summary, the mean range of B_0_ experienced in the apical myocardium due to cardiac motion is 34.3 Hz, and due to respiratory motion is 66.7 Hz. Values have been corrected for the lower gyromagnetic ratio of the phosphorus nucleus compared to the proton nucleus.
Supporting Figure 2 ‐ B_0_ variation in apical myocardium due to cardiac and respiratory motion in a **prone** position. **a** Mean(±SD) of per‐voxel B_0_ values in the apical myocardium of one subject. The B_0_ field was measured at different phases of the cardiac cycle and at three respiratory positions (inhaled, exhaled and “middle”). **b** Range of B_0_ values experienced at each cardiac phase across the three respiratory positions. The plot shows the range of the means (black) and the maximum range (red), corresponding to the first standard deviation of the distributions. In summary, the mean range of B_0_ experienced in the apical myocardium due to cardiac motion is 34.6 Hz, and due to respiratory motion is 36.1 Hz. Values have been corrected for the lower gyromagnetic ratio of the phosphorus nucleus compared to the proton nucleus.
Supporting Figure 3. Reproducibility of other parameters (validation scans). **a&b** Correlation and Bland–Altman plots of the fitted PCr amplitude of the 4^th^ (M_0_^Ctrl^ [TRiST]) and 5^th^ (M_0_^Ctrl^ [repeat]) scans. **c&d** Correlation and Bland–Altman plot of the calculated M_0_’ (from scans 2&3) and the directly measured M_0_’ (scan 6). The six different healthy subjects are shown with different marker shapes.
Supporting Figure 4 – Effect of signal‐to‐noise ratio (SNR) on the accuracy and precision of TRiST measured k_f_^CK^. Monte Carlo simulation of the TRiST measurement protocol was carried out at a range of different SNRs. The SNR was that of the PCr peak in the control scan (step 4 in Table 2). The plot was generated from 50000 repeats of the simulated measurement, with independent Gaussian noise added to each repeat for each SNR level. The plot shows the mean and standard deviation of the resulting measured k_f_^CK^.
Supporting Figure 5 – Schematic of the effect of respiratory and cardiac induced B_0_ fluctuation on steady‐state saturation of γ‐ATP. When fluctuation is low (blue line) the γ‐ATP peak remains within the amplitude modulated broadened DANTE pulse's saturation band. The peak is therefore saturated at all times and the steady‐state condition is fulfilled. If the fluctuation is large (black/red line) at some points the peak's position is shifted outside the saturation band and the steady‐state saturation condition is violated.
Supporting Table 1 – Summary of results. All values shown in this table are also contained in the results section text. Values are given as subject mean ± standard deviation. SNR is of the PCr peak in the control scan.
