# Supplementary material for: Localized rest and stress human cardiac creatine kinase reaction kinetics at 3 T
Source: NMR Biomed. 2019 Mar 28;32(6):e4085. doi: 10.1002/nbm.4085 (PMC6542687; doi:10.1002/nbm.4085)
Supplement: Supplementary file 1 — Figure S1 ‐ B0 variation in apical myocardium due to cardiac and respiratory motion in a supine position. a Mean(±SD) of per‐voxel B0 values in the apical myocardium of one subject. The B0 field was measured at different phases of the cardiac cycle and at three respiratory positions (inhaled, exhaled and “middle”). b Range of B0 values experienced at each cardiac phase across the three respiratory positions. The plot shows the range of the means (black) and the maximum range (red), corresponding to the first standard deviation of the distributions. In summary, the mean range of B0 experienced in the apical myocardium due to cardiac motion is 34.3 Hz, and due to respiratory motion is 66.7 Hz. Values have been corrected for the lower gyromagnetic ratio of the phosphorus nucleus compared to the proton nucleus. Supporting Figure 2 ‐ B0 variation in apical myocardium due to cardiac and respiratory motion in a prone position. a Mean(±SD) of per‐voxel B0 values in the apical myocardium of one subject. The B0 field was measured at different phases of the cardiac cycle and at three respiratory positions (inhaled, exhaled and “middle”). b Range of B0 values experienced at each cardiac phase across the three respiratory positions. The plot shows the range of the means (black) and the maximum range (red), corresponding to the first standard deviation of the distributions. In summary, the mean range of B0 experienced in the apical myocardium due to cardiac motion is 34.6 Hz, and due to respiratory motion is 36.1 Hz. Values have been corrected for the lower gyromagnetic ratio of the phosphorus nucleus compared to the proton nucleus. Supporting Figure 3. Reproducibility of other parameters (validation scans). a&b Correlation and Bland–Altman plots of the fitted PCr amplitude of the 4th (M0 Ctrl [TRiST]) and 5th (M0 Ctrl [repeat]) scans. c&d Correlation and Bland–Altman plot of the calculated M0’ (from scans 2&3) and the directly measured M0’ (scan 6). The six different healthy sub [file NBM-32-na-s001.docx]

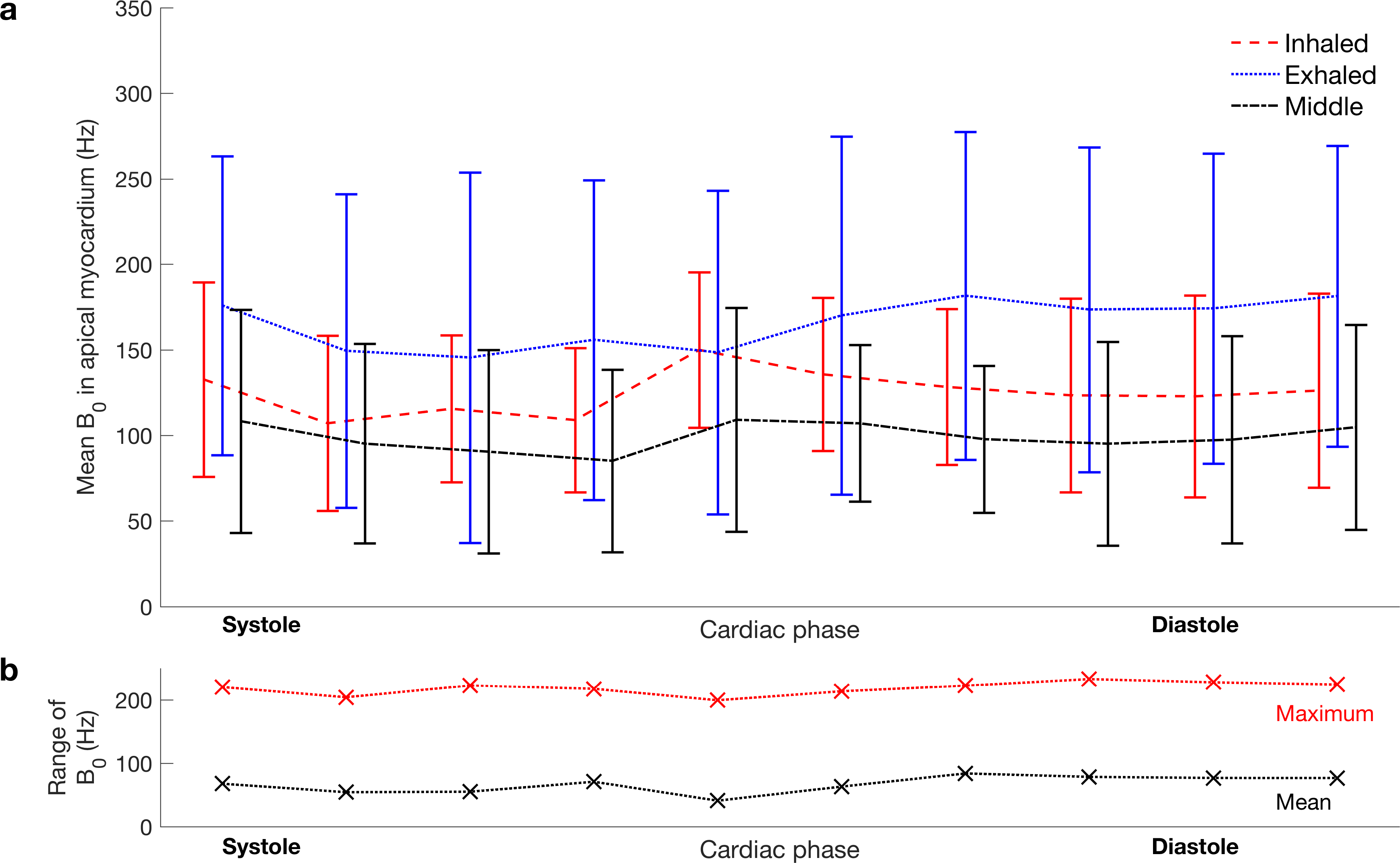


Supporting Figure 1 - B_0_ variation in apical myocardium due to cardiac and respiratory motion in a **supine** position. **a** Mean(±SD) of per-voxel B_0_ values in the apical myocardium of one subject. The B_0_ field was measured at different phases of the cardiac cycle and at three respiratory positions (inhaled, exhaled and “middle”). **b** Range of B_0_ values experienced at each cardiac phase across the three respiratory positions. The plot shows the range of the means (black) and the maximum range (red), corresponding to the first standard deviation of the distributions. In summary, the mean range of B_0_ experienced in the apical myocardium due to cardiac motion is 34.3 Hz, and due to respiratory motion is 66.7 Hz. Values have been corrected for the lower gyromagnetic ratio of the phosphorus nucleus compared to the proton nucleus.


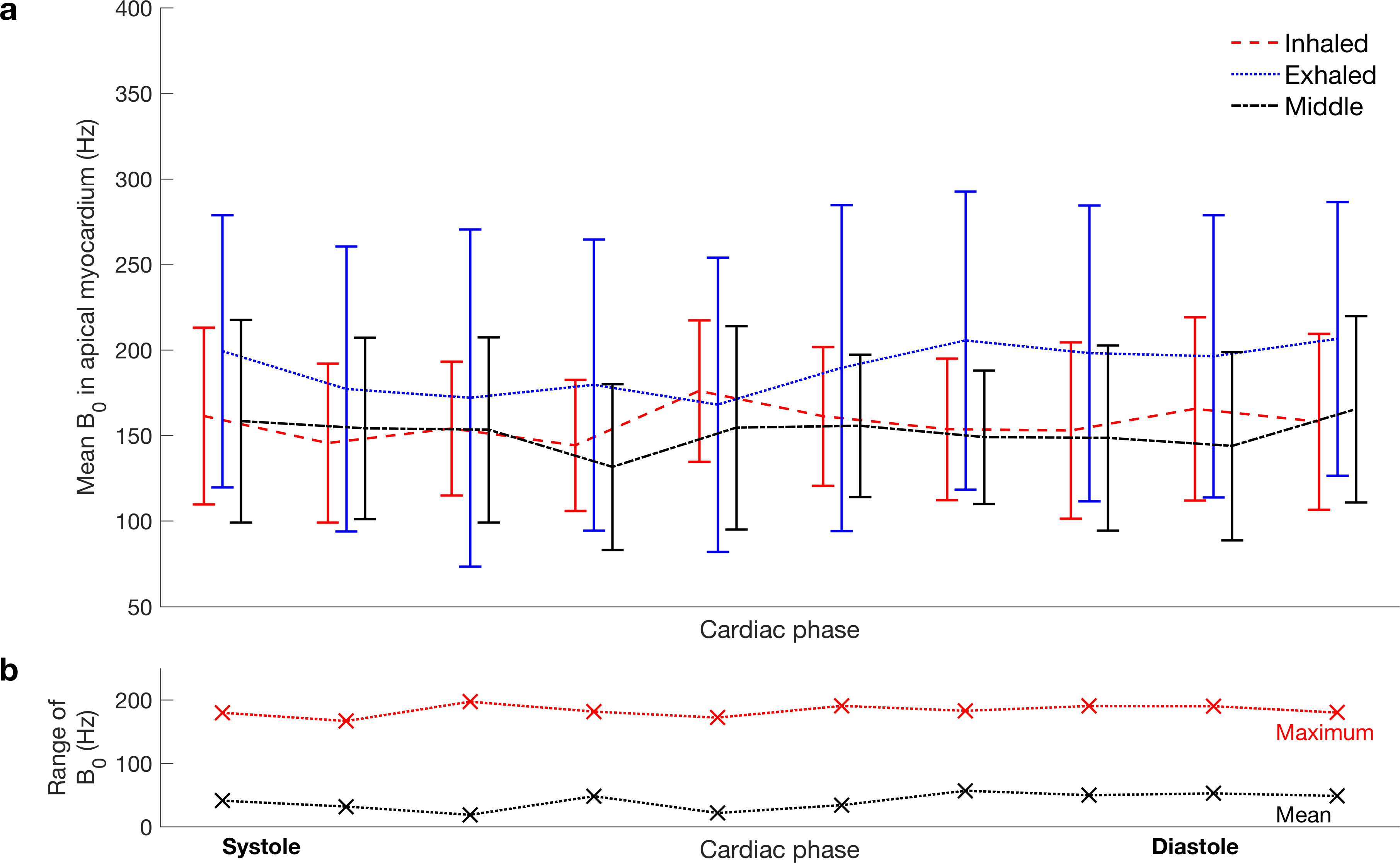


Supporting Figure 2 - B_0_ variation in apical myocardium due to cardiac and respiratory motion in a **prone** position. **a** Mean(±SD) of per-voxel B_0_ values in the apical myocardium of one subject. The B_0_ field was measured at different phases of the cardiac cycle and at three respiratory positions (inhaled, exhaled and “middle”). **b** Range of B_0_ values experienced at each cardiac phase across the three respiratory positions. The plot shows the range of the means (black) and the maximum range (red), corresponding to the first standard deviation of the distributions. In summary, the mean range of B_0_ experienced in the apical myocardium due to cardiac motion is 34.6 Hz, and due to respiratory motion is 36.1Hz. Values have been corrected for the lower gyromagnetic ratio of the phosphorus nucleus compared to the proton nucleus.


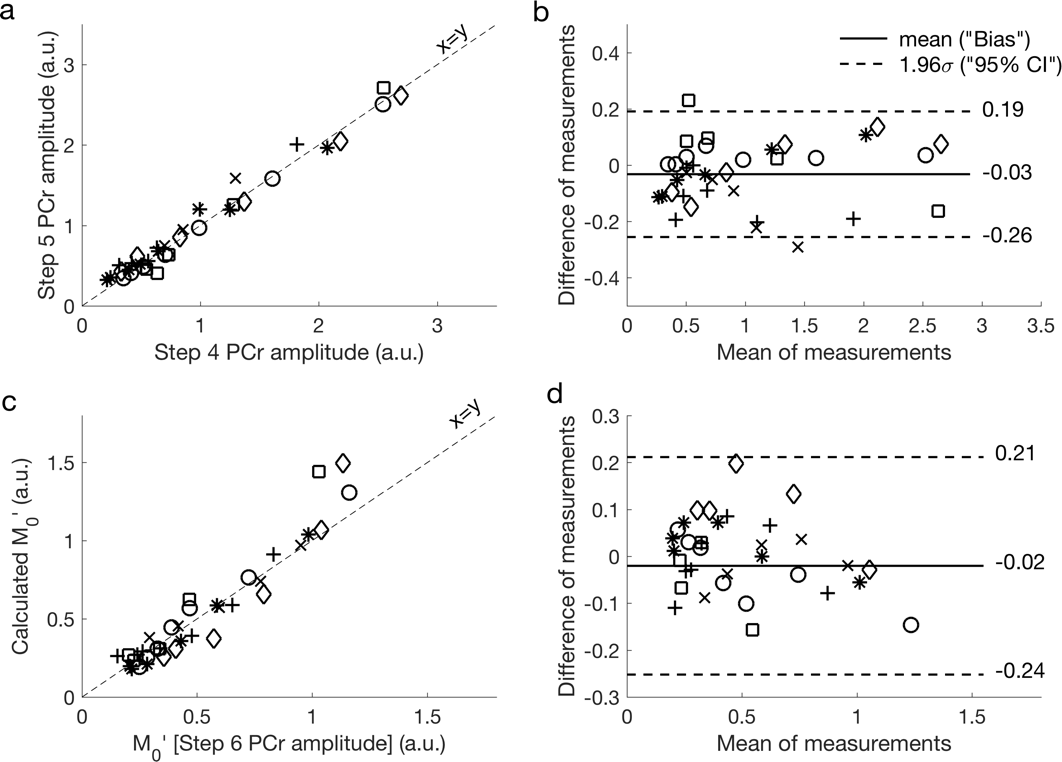


Supporting Figure 3. Reproducibility of other parameters (validation scans). **a&b** Correlation and Bland-Altman plots of the fitted PCr amplitude of the 4^th^ (M_0_^Ctrl^ [TRiST]) and 5^th^ (M_0_^Ctrl^ [repeat]) scans. **c&d** Correlation and Bland-Altman plot of the calculated M_0_’ (from scans 2&3) and the directly measured M_0_’ (scan 6). The six different healthy subjects are shown with different marker shapes.


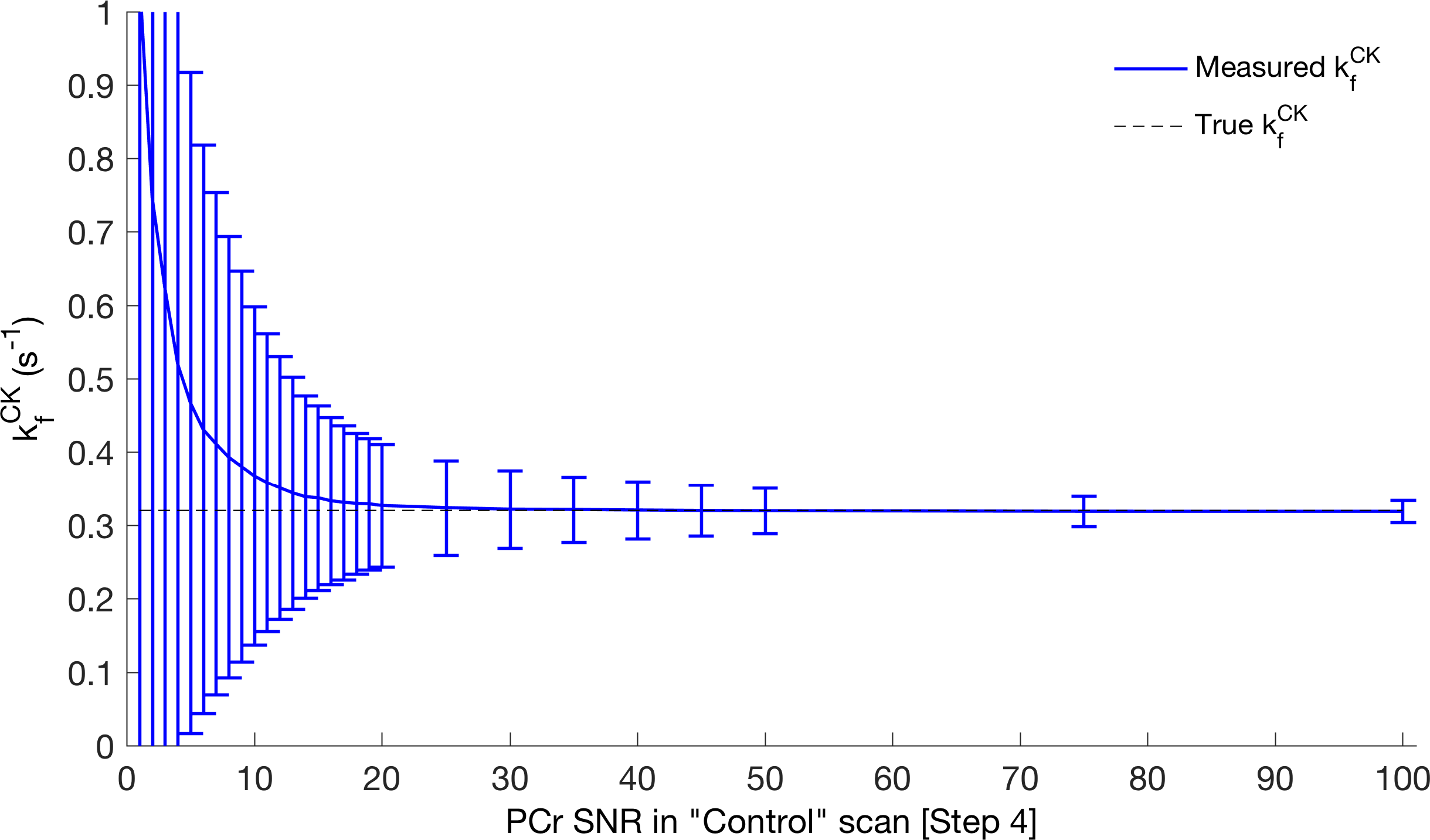


Supporting Figure 4 – Effect of signal-to-noise ratio (SNR) on the accuracy and precision of TRiST measured k_f_^CK^. Monte Carlo simulation of the TRiST measurement protocol was carried out at a range of different SNRs. The SNR was that of the PCr peak in the control scan (step 4 in Table 2). The plot was generated from 50000 repeats of the simulated measurement, with independent Gaussian noise added to each repeat for each SNR level. The plot shows the mean and standard deviation of the resulting measured k_f_^CK^.


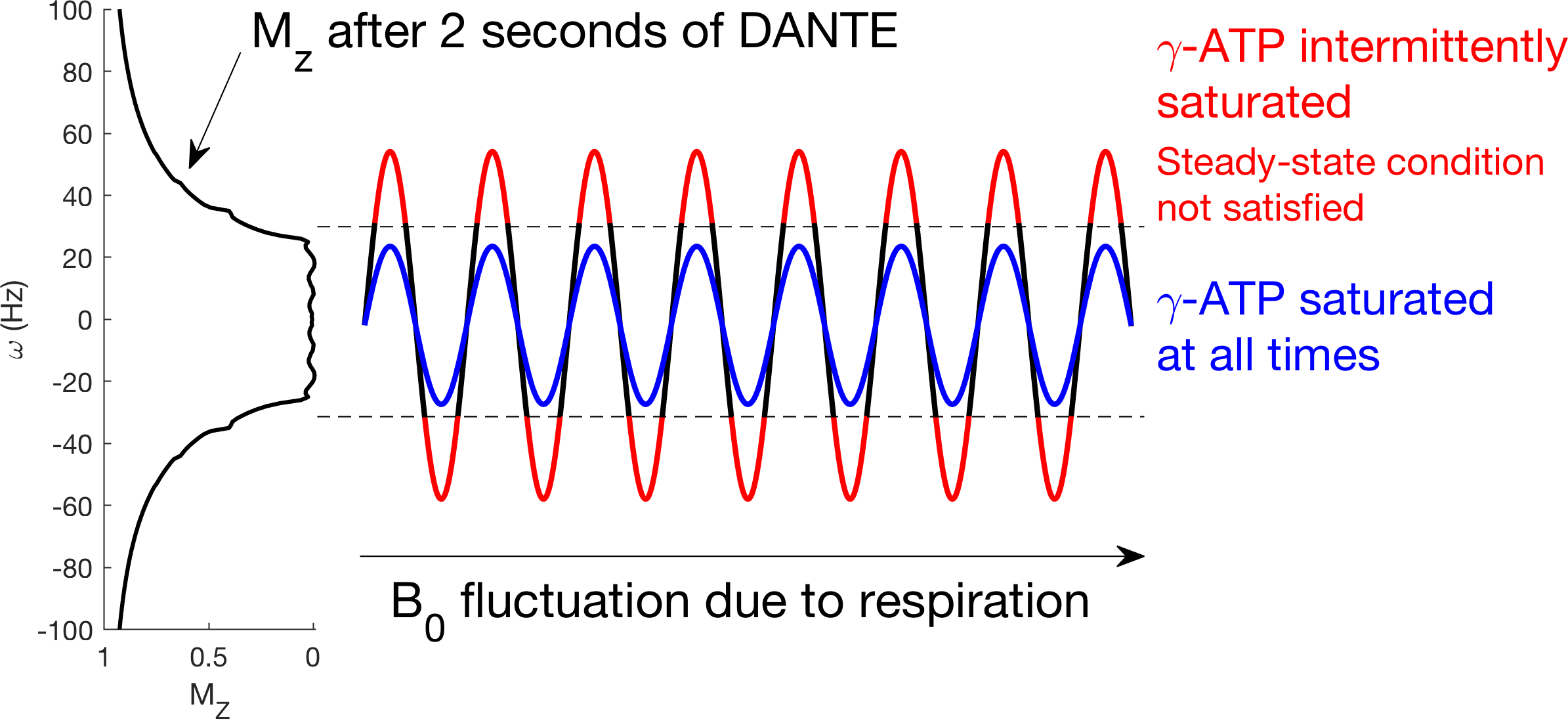


Supporting Figure 5 – Schematic of the effect of respiratory and cardiac induced B_0_ fluctuation on steady-state saturation of γ-ATP. When fluctuation is low (blue line) the γ-ATP peak remains within the amplitude modulated broadened DANTE pulse’s saturation band. The peak is therefore saturated at all times and the steady-state condition is fulfilled. If the fluctuation is large (black/red line) at some points the peak’s position is shifted outside the saturation band and the steady-state saturation condition is violated.

| Results Section | N | All cardiac slices | | | Apical slices | |
| --- | --- | --- | --- | --- | --- | --- |
|  |  | Slices | SNR | k_f_^CK^ (s^-1^) | SNR | k_f_^CK^ (s^-1^) |
| Validation of TRiST implementation  Skeletal muscle (calf) | 9 | 65 | 45±32 | 0.25±0.03 | - | - |
| Validation of TRiST implementation  Myocardium in prone position | 10 | 29 | 18 ± 8 | 0.29±0.09 | 19 ± 5 | 0.32±0.15 |
| Validation of TRiST implementation  Myocardium in supine position | 10 | 30 | 16 ± 9 | 0.15±0.10 | 17 ± 6 | 0.24±0.12 |
| Validation of MRS measured k_f_^CK^ by surgical biopsy | 25 | - | - | - | 16 ± 6 | 0.21±0.10 |
| Validation of the stress kfCK measurement (StreST) in healthy volunteers - 1^st^ Measurement | 6 | 36 | 16 ± 9 | 0.14±0.08 | 15 ± 5 | 0.18±0.08 |
| 2^nd^ Measurement |  |  |  | 0.22±0.14 |  | 0.18±0.05 |
| StreST in obese subjects and age-matched controls | 34 | 209 | 14 ± 9 | 0.12±0.08 | 15±6 | 0.16±0.08 |
| obese subjects – rest | 18 | - | - | - | - | 0.16±0.07 |
| obese subjects – stress | 18 | - | - | - | - | 0.17±0.11 |
| age-matched controls – rest | 6 | - | - | - | - | 0.15±0.09 |
| age-matched controls – stress | 6 | - | - | - | - | 0.17±0.15 |

Supporting Table 1 – Summary of results. All values shown in this table are also contained in the results section text. Values are given as subject mean ± standard deviation. SNR is of the PCr peak in the control scan.
